# An Account of Cases Shewing the Prejudicial Effects of Mercurial Vapours upon Looking-Glass Manufacturers, &c.

**Published:** 1831-11

**Authors:** Charles Mitchell

**Affiliations:** Surgeon.


### EFFECTS OF MERCURIAL VAPOURS.

*An Account of Cases shewing the Prejudicial Effects of Mercurial Vapours upon Looking-glass Manufacturers, &c.* By Charles Mitchell, Esq., Surgeon.

It is well known that persons who are employed in silvering mirrors are exposed to great hazard, from the mercurial vapours which arise during that process; but I am not aware that any particular account of the symptoms and effects produced by this cause have been given to the profession: the following cases will therefore, I trust, be worthy attention. I may premise, that the most remarkable characteristics of this mercurial influence are difficult enunciation, pain and constriction about the præcordia, emaciation, debility, and involuntary tremors. Similar effects are frequently produced in miners, gilders, and other artisans, whose trade exposes them to the operation of the same poison.

Case I. Peter Cataneo, an Italian, had worked for five years at silvering mirrors; during that time he had repeatedly been obliged to desist from his employment, until the effects of the mercury subsided.

November 1829. The tremors are general; gums sore; spirits depressed; bitter taste in the mouth, which is also very clammy; tongue white; temperature of the skin sensibly above the natural standard; pulse quick and small, but is with difficulty felt, in consequence of the constant tremor: he likewise complained of cough and tightness. He took the sulphur, as recommended by those who have practised at the mines, with some degree of benefit; a grain of opium at bedtime; and his subsistence consisted of milk, gruel, fish, and porter. He used, for the sore mouth, an acid gargle. The ptyalism abated; the tremors subsided, and in the course of a fortnight, in a great degree, vanished; leaving, however, behind a great feeling of weakness, which was successfully combated by nutritious diet and bark.

The injunctions never to resume the employment of silvering had no effect; but he has since, I understand, been obliged to relinquish it.

Case II. J. Gattoni has worked seven years, omitting one month occasionally, to allow the subsidence of any of the unpleasant symptoms which might have arisen from the influence of the mercury.

January 1831. Constant tremors; speech impeded; great salivary discharge; fœtid, loose, spongy, and ulcerated gums; face swollen; headach; slight febrile action; tongue white; appetite impaired; bowels rather confined. Haust. Aperient. ℥iss. quâque secundâ horâ donec alvus respond. ℞. Sulph. Sublimat. ʒss.; Pulv. Dover. gr. viij. M. fiat pulv. quater die sumend. ℞. Acid. Sulph. dilut. ʒijss.; Alumen, ʒi.; Aquæ, ℥xij. M. fiat garg. sæpe utend. Habeat dieta lactis.

The bowels were freely acted upon; the salivation yielded; the tremors gave way, and he rapidly recovered, and gained strength under nutrient diet, generous drinks, and muriated tincture of iron: the shaking did not, however, completely subside; neither did the faultering in the speech. The powders caused much diaphoresis, in proportion to which the tremors and febrile action abated.

In twelve looking-glass manufactories I have visited, it clearly appeared that the metal became oxidized, by combining with part of the oxygen of the atmosphere, and the more quickly so from the friction which is necessary in the application of the quicksilver to the plate of glass. Being in a state of oxide, the mercury becomes more adherent to the fingers, thereby admitting more freely of its absorption, or its insinuation beneath the nails, which is still more favorable to its dissemination throughout the system: during the friction a minute dust rises up, and affects the eyes, nose, and mouth; brings on ptyalism, renders the teeth black and crusty, causes the mouth to become clammy, and the tongue white: handkerchiefs have been applied around the mouth as a preventive of the evil; its effects were, however, uncontrollable; for the dust became more conglutinated by the moisture imbibed in the silk during respiration, and the deleterious principle of the metal appeared to have a much quicker diffusion than when no such precaution was had recourse to. Cleanliness appeared to be the best preservative in every workshop inspected: in one I found a very industrious Italian, who had worked for twenty-five years, and who was very attentive in respect to cleanliness; his constitution had never been influenced to any great extent, but he had been subjected to slight salivations at different times. During that extended period, he had been obliged to relinquish work occasionally, to permit the tightness and pain across the epigastrium, with the tremors, to subside: observance to occasional days of intermission from work were strictly adhered to, upon the least disposition to increase of the tremors or ptyalism.

Cleanliness is best effected by washing the face and hands carefully before meals, cleansing the mouth and teeth, brushing the sleeves of the coat, and likewise the hair. Certain habits should be avoided, as taking snuff, particularly when the fingers are covered with oxide; or sweeping the mercury with the flat hand from the board into the gutter which contains the metal; or applying the fingers, inadvertently or otherwise, when laying it upon the glass with the tinfoil, which renders it more adhesive to the fingers.

The gums in some of these mechanics were wasted, and not adherent to the necks of the teeth; the gums tender, and the teeth loose. An Englishman, who had suffered severely from its effects, had continual tremor, and tenderness of the gums, with mercurial fœtor: the first attack destroyed his general health; he went into the country, where, by moderate exercise, nutritious diet, chalybeates, and the warm bath, he rapidly recovered. Upon returning to the metropolis, he resumed his work, but was again soon affected, and the symptoms continued to recur at intervals; his articulation became more defective, and he was obliged to exert a great effort to render his articulation of words distinct. Finding his constitution would not permit of constant employment, he in a great measure relinquished it, and betook himself to the easier and safer part of his occupation: he is at this period remarkably irritable and peevish in his disposition.

Some men work every alternate day, so as to prevent the imbibition of the deleterious vapours. As far as I have been able to trace, the fingers and arms are first affected with tremors, then the knees, limbs, and tongue.

Drunkenness tends greatly to debilitate the system, thereby rendering it the more susceptible to the mercurial influence, which, in some instances, produces such a desperate salivation, that the patient takes his food with very great inconvenience.

I had a case of ptyalism last year: an Irishman brought from St. Bartholomew's Hospital, where he had been under treatment for hemiplegia: the pulse was quick and small; the skin hot and dry, much thirst; the tongue black, and red at the margins; destructive ulceration of the gums; a few of the teeth had fallen from their sockets, and the rest were loose; the inside of the cheeks were deeply ulcerated, and inflamed; the angles of the mouth corroded by deep fissures; very fœtid salivary discharge. Every means that could possibly be devised failed in affording the least relief, and the poor man fell a victim to a nurse's carelessness.

Some cases are attended with slight convulsive twitching, in combination with the tremulous motion, &c., a most remarkable instance of which, I subjoin.

P. Nash, ætat. twenty, of nervous temperament, commenced silvering six months ago; the trembling came on three days after he began to work, and his mouth was sore in six days: and he has continued to suffer more or less up to the present time.

14th May, 1831. The speech greatly impeded; the limbs totter when he attempts to stand or walk, which he accomplishes very slowly, with great difficulty, an infirm step, and awkward gait; he is unable to convey any substance to the mouth, in consequence of the severity of the tremors; slight subsultus tendinum, confined to the upper extremities; the tongue quivers, gums slightly tender; pulse strong, rather quick; appetite diminished; sleep disturbed; body wasted; he complains as if a feeling oppressed him like a load across the lower part of the chest; or as if a substance lay in the bottom of the lungs, as he expressed himself, which he conceived to have been drawn in by inspiration; the breathing was quick, accompanied with strictured feeling and cough. To have a warm bath and an aperient draught directly, and the following draught at bedtime: ℞. Liq. Ammon. Acet. ℥ss.; Liq. Opii Sedativ. ♏xii; Aquæ ℥j. M.

He was nearly thrown from the bath, when put into it, from the violence of the trembling; a large quantity of the water was driven by his excessive agitation over the sides of the bath; and if two men had not held him steadily in the water, he must have been thrown out before he was capable of remaining quiet. He was removed from it in ten minutes, in a very exhausted state; the body was placed in blankets, after being properly dried. Reaction followed, and he perspired freely.

15th. Pulse fuller, quick; little or no twitching; tongue clean; cough troublesome; tremors not the least abated. ℞. Sulph. Sublimat. ʒss. quater die. Rept. Haust. hora somni, c. Antim. Tart. gr. $\frac{1}{8}$.

16th. He perspired freely during the night; the tremors continue unabated; the appetite is returning; pulse quiet. Rep. med. et balneum.

17th. Complains of pain whenever he attempts to breathe deeply; the pulse has become quicker and small. App. Pect. Emp. Lyttæ.

18th. Pain and cough relieved.

19th. Complains of excessive weakness; the tremors continue unrelieved. ℞. Tinct. Mur. Ferr. ♏x. quater die; to have four ounces of animal food, and a glass of wine, daily.

20th. A sensible diminution in the severity of the trembling. Perstet.

We were soon able to increase the allowance of food, as the affection of the chest did not prove troublesome; neither did local inflammations arise elsewhere, a very common occurrence in cases of rapid repletion, subsequent upon excessive states of debility. Upon the tremors susiding he was sent to Margate, where he resided a month, with great benefit to his general health. During his stay there he took the carbonate of iron.

*Lambs' Conduit street.*

Some interesting remarks are made respecting the injurious effects of the mercurial vapours, to which looking-glass manufacturers and other artisans are exposed, by M. MERAT, in the Dict. des Sc. Med., tom. 30, p. 232.* M. Merat, and other French writers, designate the disease by the term of *Tremblement des doreurs:* his description of the symptoms is very similar to that given by Mr. MITCHELL. He states that metal gilders and some other artisans, who use an amalgam of gold or silver with mercury, are subject to violent trembling, which almost amounts to convulsions; the arms are particularly affected. This trembling is sometimes general, and occasionally so violent that the patient is obliged to be fed; for, on account of the want of command over, and the great irregularity and sudden violence of the muscular actions, the face is frequently injured when an attempt is made to raise the hand to the mouth. M. Merat does not consider this affection very dangerous; but he says that the workmen are often obliged to abandon their employment: he recommends the mode of treatment adopted by Mr. Mitchell. "The mercurial trembling is cured by quitting work; residence in a pure air; the use of warm baths, sudorifics, and antispasmodics. Severe relapses are frequent, and each attack is usually longer than the preceding, and more difficult to relieve. The workmen are sometimes affected with the trembling for months together, particularly during winter, at which season such attacks are more frequent and more difficult to cure."

Dr. CHRISTISON† may also be consulted with advantage upon this subject: he mentions the disease under the term of shaking palsy, or mercurial trembling, and states that it is not merely long-continued exposure to mercurial preparations that causes it. A single strong exposure may be sufficient; and the same exposure may cause tremens in one, and salivation in another. Mr. HAIDINGER related an accident to Dr. Christison, that occurred to a barometer maker of his acquaint-

* The same author also gives an instructive account of the *tremblement metallique,* in an Appendix to his "Traité de la Colique Metallique."

† On Poisons, p. 310.

ance, which illustrates both these statements. This man and one of his workmen were exposed one night, during sleep, to the vapours of mercury, from a pot on a stove in which a fire had been accidentally kindled: they were both most severely affected; the latter with salivation, which caused the loss of all his teeth; the former with shaking palsy, which lasted his whole life.

The precautions which are suggested by Mr. Mitchell, and others who have written upon the same subject, will no doubt generally prevent the workmen from suffering from the lamentable effects of exposure to mercurial and other deleterious vapours; but more effectual means of protection have been devised, and it is by no means creditable to the masters of manufactories in which such dangers exist that they are not always adopted. If their own humanity will not induce them to take advantage of every suggestion to protect artisans from the many dangers that are incidental to their employments, the authority of government should be exerted in their defence. Mr. Thackray* most truly observes, that an examination into the state of our manufactures has long been demanded, alike by humanity and by science; and he affords abundant proof of the power which the master manufacturer frequently possesses, but which he *rarely exerts*, of not only ensuring the health and safety of the "operatives," but of adding to their comfort and diminishing their vices.

A philanthropic merchant of Paris, M. Ravrio, who had amassed a considerable fortune in trade as a gilder, and who had frequently witnessed the dangers to which his workmen were exposed, left by his will three thousand francs to be given to any person who should devise the best means of protecting them against the hazards of their employment. The prize was awarded by the Academy of Science to M. Darcet, who invented a very simple, yet efficacious, means of diminishing the ill effects which arise from exposure to mineral and other deleterious vapours. For an account of M. Darcet's contrivance, we refer our readers to the Dict. des Sc. Med., tom. 30, p. 234, art. "*Doreurs sur Metaux*," and tom. 27, p. 298, art. "*Latrines*."—Editor.

* On Employments, &c.

